# Housing Insecurity among Emergency Department Patients with Opioid Use Disorder

**DOI:** 10.5811/westjem.25025

**Published:** 2025-11-17

**Authors:** Christine M. Shaw, Whitney Covington, Lauren A. Walter

**Affiliations:** University of Alabama at Birmingham, Department of Emergency Medicine, Birmingham, Alabama

## Abstract

**Introduction:**

Emergency departments (ED) have increasingly engaged in screening and treatment initiation for patients with opioid use disorder (OUD). Patients with OUD, however, may also be impacted by significant social need, including housing insecurity. We sought to consider the incidence of homelessness and housing insecurity in patients engaged in an ED-initiated medication for opioid use disorder (MOUD) program.

**Methods:**

We performed a secondary analysis, with specific consideration of housing status, on data obtained from a prospective, ED-initiated MOUD study conducted at an urban, academic hospital, inclusive of enrollments from July 2019–February 2022. We obtained data from participant interviews conducted at study intake and at three months to include the question: “In the past 30 days, where have you been living most of the time?” We used descriptive statistics and Pearson chi-square analyses to assess the data.

**Results:**

Of 315 participants, most were White (79.4 %), male (64.4 %), and between the ages of 25–44 (74.6%). At intake, 66 (20.9%) reported active homelessness, including 44 (14.0%) unsheltered. An additional 157 (49.8%) met criteria for housing insecurity. Men were more likely to be experiencing homelessness (25.1% men reported homelessness vs 13.4% women, *P =* .01). In contrast, women trended toward housing insecurity more than their male counterparts (45.8% men with housing insecurity vs 57.1% women, *P =* .05). At three-month follow-up, 141 were able to be reached, with a predominance of housed individuals (118 housed; 46.8%); in contrast only 34.8% of persons experiencing homelessness) (23 participants) were able to follow up at three months (*P =* .07). Significant differences between sexes noted at intake resolved. No significant differences were found at intake or three months when considering race or age comparisons.

**Conclusion:**

Patients in the ED who are engaged in care for OUD are disproportionately (70.8%) impacted by homelessness and housing insecurity; further, sex may play an exacerbating role. Emergency department-initiated MOUD treatment may have a positive impact on housing status, suggested by this study; however, the study was limited due to large loss to follow-up, especially among those with housing insecurity.

## INTRODUCTION

In response to the worsening opioid epidemic, emergency clinicians and emergency departments (ED) have increasingly engaged in screening, brief intervention, and treatment initiation for opioid use disorder (OUD).^1^ Opioid-related ED visits have nearly tripled in recent years. Numerous studies indicate that starting OUD treatment for patients while they are still in the ED significantly raises the chances of subsequent treatment retention^2^ and is associated with abstinence from illicit drug use and improved quality of life.^3^

Homelessness is yet another growing public health concern in the United States (US). Most recent point-in-time counts suggest that well over half a million persons in the US are experiencing homelessness on any given night.^4^,^5^ The ED plays a significant role for this cohort. The rate of persons experiencing homelessness who use the ED, often as a safety-net healthcare resource, has more than doubled in the past decade.^6^ Persons experiencing homelessness often face significant barriers to routine medical care, which contributes to increased ED use.^7^ Homelessness and housing insecurity are frequent social comorbidities associated with substance use disorder and OUD. Study reports vary but suggest that 16–50% or more of this cohort suffer from a substance use disorder. The overlap and bidirectional association between homelessness and opioid use, in particular, can be quite profound.^8^,^9^

Although ED-initiated screening and treatment programs for OUD have generally demonstrated positive impact, it is unclear how they might impact persons experiencing homelessness specifically.^9^ Housing-insecure patients may have economic, transportation, or other situational barriers that could make post-ED treatment adherence and definitive linkage challenging.^10^ Further, while acute opioid withdrawal or opioid overdose presentations may make OUD relatively easier to identify, homelessness and/or housing insecurity might be more challenging for a clinician to ascertain unless the question is explicitly asked and answered.^11^ Finally, little is known about how ED-initiated OUD treatment might affect housing status. Thus, we sought to characterize the incidence of homelessness and housing insecurity in patients engaged in an ED-initiated medications for opioid use disorder (MOUD) program; we further aimed to consider the association of ED-initiated OUD treatment with housing status over time. A better understanding of the impact of this particular social need among this cohort could guide future interventions in the ED and public health space.

**Population Health Research Capsule**

What do we already know about this issue?

*Homelessness and housing insecurity are frequently associated with opioid use disorder (OUD), and ED-related visits have nearly tripled.*

What was the research question?

*What is the incidence of homelessness and housing insecurity in patients engaged in an ED-initiated medication for opioid use disorder program?*

What was the major finding of the study?

*70.7% of people with OUD presenting to the ED have housing insecurity or are homeless (P = .01).*

How does this improve population health?

*This study provides evidence that persons with OUD disproportionately struggle with housing insecurity and suggests clinicians should consider this social risk.*

## METHODS

We performed a secondary analysis of previously collected data from a prospective study. The primary study objective was to identify ED patients impacted by OUD, initiate them on MOUD from the ED, and link them to definitive care. To better understand the impact of specific social determinants of health on this cohort, we focused on study participants more specifically by housing status in this secondary analysis.

The ED-based MOUD initiation program was conducted from July 2019–February 2022, at the University of Alabama Birmingham (UAB) University Hospital. The UAB Institutional Review Board reviewed and approved this study. The UAB Hospital is an urban, academic ED with a volume of > 75,000 annual patient visits. The ED-based MOUD program was supported by the Substance Abuse and Mental Health Services Administration (SAMHSA, ¶H79TI081609), and a detailed protocol has been previously described.^11^ Patients presenting to the ED with a primary complaint of non-fatal opioid overdose, opioid withdrawal, requesting opioid detoxification, or otherwise meeting *Diagnostic and Statistical Manual of Mental Disorders, 5^th^ Ed*, criteria for moderate to severe OUD, were considered for study inclusion, following medical stabilization and clearance. Emergency physicians engaged potential enrollees in a brief, negotiated interview to confirm OUD diagnosis and to assess motivation to begin treatment for OUD. A subsequent physician-activated order in the electronic health record notified research staff, 24/7, of a physician-confirmed eligible patient. Exclusion criteria included patients who were already actively engaged in a MOUD treatment program, and those who were medically or psychiatrically unstable, unable to consent, or otherwise considered to be part of a vulnerable population (eg, pregnant; incarcerated/in police custody). Research staff conducted enrollment and assisted with linkage to follow-up care. Emergency physicians provided a 10-day prescription of buprenorphine/naloxone at time of enrolled patient discharge to bridge the patient pharmaceutically until follow-up appointment. As required by SAMHSA Government Performance and Results Modernization Act (GPRA) assessments, our research staff collected comprehensive patient-specific information including general demographics, substance use and misuse information, as well as mental health and physical health quality of life variables, at time of enrollment and by a community tracking service agency at three-months post-enrollment (SAMHSA, 2019); this included a housing-specific GPRA variable: “In the past 30 days, where have you been living most of the time?” Follow-up responses were most typically solicited via phone although the tracking agency also facilitated in-person meetings for data capture via in-person shelter visits, for instance. Responses were characterized as a) securely housed (eg, own apartment, room, trailer or house); b) housing insecure (eg, someone else’s apartment, room, trailer or house, hotel or motel, halfway house or transitional housing); c) sheltered homeless; or d) unsheltered homeless. Unsheltered homelessness was defined as sleeping on the streets, outdoors in a tent, in a car, or any other location not intended for human habitation.

General descriptive statistics were considered, and we used the Pearson chi-square test for comparative analyses. We used JMP Pro 17 (SAS Institute Inc, Cary, NC) for all statistical analyses, and statistical tests were performed at ¶ = 0.05 significance level. Data organization and analysis were conducted with consideration of the Strengthening the Reporting of Observational Studies in Epidemiology guidelines.^12^

## RESULTS

During the study time frame, 866 patients presented for OUD-related complaints: 551 (63.6%) did not meet eligibility criteria, and 315 ultimately completed enrollment. The majority of those enrolled were White (79.4 %), male (64.4 %), and 25–44 years of age (74.6 %) (Table 1). Of note, 78.1% of screen fails were White; sex of screen fails was unavailable. At the time of enrollment, overall reported rates of homelessness and housing insecurity were 20.9% and 49.8%, respectively, accounting for most (70.8%) of the participants. Men (25.1%) were more frequently observed to be experiencing homelessness at enrollment than women (13.4%; *P =* .01). No significant differences were noted in housing-specific demographics with regard to age at time of enrollment. All persons experiencing homelessness were more frequently observed to report unsheltered homelessness (66.7%) as compared to sheltered. Residing in someone else’s apartment, trailer, or house (70.7%) was the most common form of housing insecurity reported, more frequently by women (53.5%) and younger participants (78.3%).

At three months’ post ED-enrollment, 141 (44.8%) participants were able to be reached for repeat GPRA assessment, including housing demographic variable inquiry (Table 2). At three months, the incidence of persons experiencing homeless decreased to 7.8%, although housing insecurity (45.4%) remained relatively unchanged. Most of these persons (63.6%) remained unsheltered. Significant demographic differences noted in sex at intake did not persist at three months. Younger participants, 18–24 years of age, continued to report high rates of housing insecurity, namely living in someone else’s apartment, room, trailer, or house, at three months.

When considering those who reported homelessness at intake specifically, 23 (34.8%) were able to be reached for three-month assessment, including 16 men (31.3% of initial male cohort) and seven women (46.7% of initial female homeless cohort), and 20 White participants (35.1% of initial White homeless cohort), two Black participants (28.6% of initial Black cohort). This is in comparison to a 47.4% three-month follow-up rate for those not experiencing homelessness at intake. Of those 23 reporting being homeless at intake who completed three-month assessment, 17 (73.9 % or 25.8% of initial cohort) reported now being housed, including 12 (75.0%) men and five (71.4%) women; three reported remaining in a shelter (two men, one woman), and three remained unsheltered (two men and one woman). In comparison, 171 persons did not follow up, which included 139 White (80.0%), 120 men (69.0%); no statistical difference between groups was found. Twenty-three (34.8%) of the initial 66 persons experiencing homelessness were able to be reached for three-month assessment, including 16 men (31.3% of initial male homeless cohort) and 7 women (46.7% of initial female homeless cohort), and 20 White (35.1% of initial White homeless cohort), 2 Black (28.6% of initial Black homeless cohort) (one “other”-Hispanic). This is in comparison to a 47.4% three-month follow-up rate for those not experiencing homelessness at intake. While a follow-up trend is apparent, favoring those not experiencing homelessness at intake, it did not reach statistical significance (*P* = .07).

Of note, since intake, one male participant went from previously characterized as “housed” to residing in a shelter, and four women went from “housed” to unsheltered (all White). Each of these individuals were classified as “housing insecure” at intake (four residing in someone else’s place and one [female] residing in a hotel).

## DISCUSSION

Emergency departments serve as the primary healthcare setting for a significant portion of persons experiencing homelessness in the US, constituting over a half million ED visits per year.^13^ Intake housing demographics in this study underscore this relationship and highlight the marked overlap between OUD and homelessness in particular; one in five participants in this OUD-focused intervention was experiencing homelessness at time of study enrollment, an incidence much higher than the range of 0.5–13.8% of persons experiencing homelessness among all ED patients reported in prior studies.^14^ While the local overlap of the incidence of homelessness and ED use is unclear, in our study time frame the incidence of homelessness we noted was also higher than local reports, which note that approximately 15% of the population of Jefferson County (location of UAB) were living with severe housing insecurity issues in 2021.^4^ Further, most of the persons experiencing homelessness in this review were unsheltered, primarily living on the streets; this may be in large part due to sobriety requirements imposed by local shelters and transitional housing options.^15^ This incidence of social need highlights the potential role and impact of the ED in this cohort.

The male-dominated incidence of persons experiencing homelessness in this study was consistent with national trends; however, the sex-specific difference was inflated. The 2022 Alabama point-in-time count found that women accounted for 43.5% of homeless and men accounted for 55.9%, in contrast to 22.7% and 77.3%, respectively, in this intake cohort. Locally, in Jefferson County the 2021 point-in-time count found that women accounted for 28% of persons experiencing homelessness and men accounted for 72%, in relative alignment with our study percentages.^4^ This somewhat elevated unmet housing need in men with OUD, however, suggests that there may be additional sex-specific variables impacting housing access in this population specifically. These variables may be related to social support networks, which may be more limited in men, and/or prior criminal justice system involvement or concomitant externalizing mental health issues, both of which may contribute to homelessness and are more common in men.^16^,^17^ From a racial perspective, while the 2022 point-in-time count in Alabama revealed that 57.3% of homeless were Black and 37.6% were White in this study, most (86.4%) were White. This difference is reflective of the local demographics of the opioid epidemic.^18^ Younger participants were more frequently observed to be categorized as “housing insecure,” specifically to be living with someone else, although this may be most reflective of age rather than a true social determinant in this younger subgroup.

Housing status at three months is interesting to consider; notably, the incidence of homelessness decreased. A quarter of those previously experiencing homelessness were housed at three months. This may be due to a number of conjectured reasons: 1) stabilization through treatment resulting in improvement in overall well-being, to include increased stability in housing situations;^3^ 2) access to supportive services such as housing assistance programs and case management; 3) improved interpersonal functioning and employment opportunities; 4) reduction in high-risk behaviors such as criminal behaviors; 5) reintegration into supportive networks and reconnection with family, friends, and community resources; 6) and enhanced self-efficacy and coping skills, enabling one to better address housing-related issues and maintain stable living situations. Initiating treatment for OUD addresses both the substance use itself and the underlying factors that contribute to homelessness.^19^ By providing a venue and initiating a path for definitive care, ED-initiated treatment for OUD may empower individuals to work toward achieving stable housing alongside long-term recovery.

However, while the incidence of persons experiencing homelessness decreased at three-month follow-up, housing insecurity among this cohort remained relatively elevated and unchanged. This may represent the time it takes to acquire “own” housing; three months may not be sufficient, particularly in a cohort that is often dealing with multiple social and interpersonal hurdles. Further, there were actually several persons, mostly women, who transitioned from “housed” at intake (albeit housing insecure) to experiencing unsheltered homelessness at three-month follow-up. This may suggest that the participants’ prior housing situation may have been associated with or actually been contingent upon an active substance misuse network; subsequent participation in OUD treatment may have complicated this housing relationship as the participant sought drug-free housing options, resulting in homelessness. This subgroup, in addition to those others in this cohort who continued to experience homelessness, might benefit most from a “Housing First” model approach.

Housing First is an evidence-backed strategy that focuses on securing permanent housing for individuals experiencing homelessness, especially those with complex needs like SUD or mental health challenges. This approach does not require individuals to participate in treatment or achieve abstinence before receiving housing. This model posits that providing a person with housing first, alongside or prior to OUD treatment, creates a foundation on which the process of recovery can begin.^20^ This type of model has demonstrated that following concomitant housing provision and treatment participation, participants are able to successfully obtain and maintain independent housing while decreasing or eliminating substance use and improving health outcomes.^21^ An ED-based intervention that address *both* OUD and housing issues simultaneously may be beneficial to those persons experiencing both.

## LIMITATIONS

This study was conducted at a single site and, thus, results may not be generalizable to all locations or populations. In the design of this study, as a secondary analysis, the reviewers were not blinded to the study hypothesis. Further, while not statistically significant, the relatively high drop-out rate, particularly of persons experiencing homelessness, at three-month follow-up may have led to selection bias and, therefore, may have limited our interpretation of follow-up data. Additionally, the demonstrated trend of reduced homelessness at three-month follow-up may reflect improved capacity to contact persons no longer experiencing homelessness, resulting in additional selection bias. This high loss of follow-up among PEH is consistent with previously published studies.

In addition, our analysis did not find a difference between those who followed up and those who did not. As noted, follow-up surveys were most frequently obtained via phone, occasionally in person. This decreased follow-up is anticipated as people struggling with housing insecurity or homelessness in addition to OUD often also have unreliable forms of communication due to associated socioeconomic factors, as described earlier in this paper. Consistent with all survey-based data, participant responses are also subject to reporting bias. Finally, it warrants noting that the project time frame is inclusive of the COVID-19 pandemic, which may have impacted the study population and their associated demographics.

## CONCLUSION

This study adds to the existing evidence that persons with substance use disorders such as opioid use disorder, who use the ED, disproportionately struggle with homelessness and housing insecurity; further, while deserving of additional causal-focused consideration, it may also suggest a potential impact of ED-initiated treatment for OUD on housing status. Emergency clinicians engaging in treatment initiation for OUD should consider that this social risk may be present in their patients and consider concomitant interventions, if available, to address both issues. Future acute-care interventions with focus on the opioid epidemic should consider deliberate inclusion of concomitant housing pathways or intercessions alongside OUD treatment initiation.

## Figures and Tables

**Figure 1:**
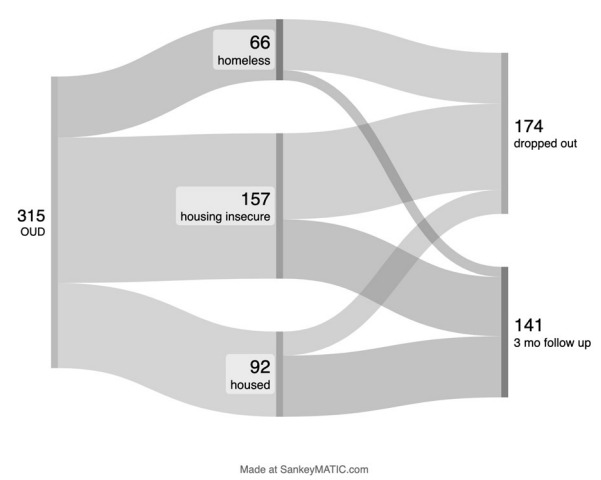
Plot of participants enrolled in a medication for opioid use disorder program and 3-month dropout vs follow- up rate. *At 3 months, 11 participants remain homeless, 64 with housing insecurity, 66 housed. *OUD*, opioid use disorder; *mo*, month.

**Table 1:** Intake housing-based demographics of patients engaged in a medication for opioid use disorder program initiated in the emergency department.

Demographic	Persons Experiencing Homelessness (PEH)*	At Risk/Housing Insecure (excludes PEH)	ALL PEH and Housing Insecure	PEH comparison P-value	Housing Insecure comparison P-value	ALL PEH and Housing Insecure comparison P-value
Total N = 315	66 (20.9%)	157 (49.8%)	223 (70.8%)			
Sex						
Male (n = 203; 64.6%)	51 (25.1%) 16 (7.9)%) sheltered 35 (17.2%) unsheltered	93 (45.8%) 82 (40.4%) Someone else’s apartment, room, trailer, or house 6 (3.0%) Hotel/motel 5 (2.5%) Halfway house/Transitional housing	144 (70.9%)	.01	.05	.94
Female (n = 112; 35.6%)	(13.4%) 6 (5.4%) sheltered 9 (8.0%) unsheltered	(57.1%) 60 (53.5%) Someone else’s apartment, room, trailer, or house 4 (3.5%) Hotel/motel	79 (70.5%)			
Race^m^,^n^						
Black (n = 58; 18.4%)	7 (12.1%) 2 (3.4%) sheltered 5 (8.6%) unsheltered	(51.7%) 29 (50.0%) Someone else’s apartment, room, trailer, or house 1 (3.3%) Hotel/motel	37 (63.8%)	.07	.57	.50
White (n = 250; 79.4%)	57 (22.8%) 19 (7.6%) sheltered 38 (15.2%) unsheltered	119 (47.6%) 110 (44.0%) Someone else’s apartment, room, trailer, or house 9 (3.6%) Hotel/motel 6 (2.4%) Halfway house/Transitional housing	171 (68.4%)			
Other (n = 5; 1.6%)	2 (40.0%) 1 (20.0%) sheltered 1 (20.0%) unsheltered	2 (40.0%) 2 (40.0%) Someone else’s apartment, room, trailer, or house	4 (80.0%)			
Age^s^ 18–24 (n = 23; 7.3%)	1 (4.3%)	18 (78.3%)	19 (82.6%)	.44^t^	.32^t-^	.19^v^
	1 (4.3%) sheltered	17 (73.9%) Someone else’s apartment, room, trailer, or house 1 (4.3%) Halfway house/Transitional house				
25–34 (n = 105; 33.3%)	20 (19.0%) 7 (6.7%) sheltered 13 (12.4%) unsheltered	58 (55.2%) 52 (49.5%) Someone else’s apartment, room, trailer, or house 4 (3.8%) Hotel/motel 1 (1.0%) Halfway house/Transitional house	78 (74.3%)			
35–44 (n = 130; 41.3%)	34 (26.2%) 11 (8.5%) sheltered 23 (17.7%) sheltered	60 (46.2%) 52 (40.0%) Someone else’s apartment, room, trailer, or house 5 (3.8%) Hotel/motel 3 (2.3%) Halfway house/Transitional house	94 (72.3%)			
45–54 (n = 40; 12.7%)	9 (22.5%) 3 (7.5%) sheltered 6 (15.0%) unsheltered	18 (45.0%) 16 (40.0%) Someone else’s apartment, room, trailer, or house 1 (2.5%) Hotel/motel 1 (2.5%) Halfway house/Transitional house	27 (67.5%)			
55–64 (n = 13; 4.1%)	2 (15.4%) 2 (15.4%) unsheltered	4 (30.8%) 4 (30.8%) Someone else’s apartment, room, trailer, or house	6 (46.2%)			
>65 (n = 4; 1.3%)	0 (0)	1 (25.0%) 1 (25.0%) Someone else’s apartment, room, trailer, or house	1 (25.0%)			

* Missing data: one “don’t know”; one “refused”;

m two “declined/refused to answer;”

n “other” excluded from statistical analyses;

s > 65 excluded from statistical analyses;

T 18–24 and 55–64 age categories excluded;

v 18–24 age category excluded.

m two “declined/refused to answer;”

n “other” excluded from statistical analyses.

**Table 2:** Demographics by housing status at three-month follow-up.

Demographic	Persons Experiencing Homelessness (PEH)	At Risk/Housing Insecure (excludes PEH)	ALL PEH and Housing Insecure	PEH comparison P-value	Housing Insecure comparison P-value	ALL PEH and Housing Insecure comparison P-value
Total N = 141	11 (7.8%)	64 (45.4%)	75 (53.2%)			
Sex						
Male (n = 83; 58.9%)	5 (6.0%) 3 (3.6%) sheltered 2 (2.4%) unsheltered	41 (49.4%) 40 (48.2%) Someone else’s apartment, room, trailer, or house 1 (1.2%) Hotel/motel	46 (55.4%)	.35	.25	.54
Female (n = 58; 41.1%)	6 (10.3%) 1 (1.7%) sheltered 5 (8.6%) unsheltered	23 (39.7%) 22 (37.9%) Someone else’s apartment, room, trailer, or house 1 (1.7%) Hotel/motel				
Race^						
Black (n = 31; 22.0%)	2 (6.5%) 2 (6.5%) unsheltered	15 (48.4%) 15 (48.4%) Someone else’s apartment, room, trailer, or house	17 (54.8%)	-	.76	.84^a^
White (n = 106; 75.2%)	8 (7.5%) 3 (2.8%) sheltered 5 (4.7%) unsheltered	48 (45.3%) 46 (44.7%) Someone else’s apartment, room, trailer, or house 2 (1.9%) Hotel/motel	56 (52.8%)			
Other (n = 3; 2.1%)	1 (33.3%) 1 (33.3%) sheltered	1 (33.3%) 1 (33.3%) Someone else’s apartment, room, trailer, or house	2 (66.7%)			
Age^s^						
18–24 (n = 7; 5.0%)	0 (0)	6 (85.7%) 6 (85.7%) Someone else’s apartment, room, trailer, or house	6 (85.7%)	-	.60^b^	.89
25–34 (n = 53; 37.6%)	5 (9.4%) 3 (5.7%) sheltered 2 (3.8%) unsheltered	22 (41.5%) 22 (41.5%) Someone else’s apartment, room, trailer, or house	27 (50.9%)			
35–44 (n = 51; 36.2%)	4 (7.8%) 1 (2.0%) sheltered 3 (5.9%) unsheltered	22 (43.1%) 20 (41.7%) Someone else’s apartment, room, trailer, or house 2 (4.2%) Hotel/motel	26 (50.9%)			
45–54 (n = 21; 14.8%)	2 (9.5%) 2 (9.5%) unsheltered	10 (47.6%) 10 (47.6%) Someone else’s apartment, room, trailer, or house	12 (57.1%)			
55–64 (n = 7; 5.0%)	0 (0)	4 (51.7%) 4 (51.7%) Someone else’s apartment, room, trailer, or house	4 (57.1%)			
>65 (n = 2; 1.4%)	0 (0)	0 (0)	0 (0)			

Missing Data:

^ one “unknown” race;

a “other” excluded from statistical analysis;

b 18–24, 55–64, and > 65 excluded from statistical analysis.

## References

[1] Thomas CP, Stewart MT, Tschampl C (2022). Emergency department interventions for opioid use disorder: a synthesis of emerging models. J Subst Abuse Treat.

[2] D’Onofrio G, O’Connor PG, Pantalon MV (2015). Emergency department-initiated buprenorphine/naloxone treatment for opioid dependence: a randomized clinical trial. JAMA.

[3] Carroll C, Hand D, Covington W (2023). Emergency-department initiated buprenorphine: Impact on quality of life. Drug Alcohol Depend Rep.

[4] (2022). The 2022 Annual Homeless Assessment Report (AHAR) to Congress.

[5] Yamamoto A, Needleman J, Gelberg L (2019). Association between homelessness and opioid overdose and opioid-related hospital admissions/emergency department visits. Soc Sci Med.

[6] (2023). QuickStats: Rate of Emergency Department Visits, by Homeless Status — National Hospital Ambulatory Medical Care Survey, United States, 2010–2021. Morb Mortal Wkly Rep.

[7] Stafford A, Wood L (2017). Tackling health disparities for people who are homeless? Start with social determinants. Int J Environ Res Public Health.

[8] McLaughlin MF, Li R, Carrero ND (2021). Opioid use disorder treatment for people experiencing homelessness: a scoping review. Drug Alcohol Depend.

[9] Doran KM, Fockele CE, Maguire M (2022). Overdose and homelessness—why we need to talk about housing. JAMA Netw Open.

[10] Cernadas A, Fernández Á (2021). Healthcare inequities and barriers to access for homeless individuals: a qualitative study in Barcelona. Int J Equity Health.

[11] Walter LA, Li L, Rodgers JB (2021). Development of an emergency department-based intervention to expand access to medications for opioid use disorder in a Medicaid non-expansion setting: protocol for engagement and community collaboration. JMIR Res Protoc.

[12] von Elm E, Altman DG, Egger M (2008). The Strengthening the Reporting of Observational Studies in Epidemiology (STROBE) statement: guidelines for reporting observational studies. J Clin Epidemiol.

[13] Ku BS, Scott KC, Kertesz SG (2010). Factors associated with use of urban emergency departments by the U.S. homeless population. Public Health Rep.

[14] Salhi BA, White MH, Pitts SR (2018). Homelessness and emergency medicine: a review of the literature. Acad Emerg Med.

[15] Schinka JA, Casey RJ, Kasprow W (2011). Requiring sobriety at program entry: impact on outcomes in supported transitional housing for homeless veterans. Psychiatr Serv.

[16] Caetano SC, Silva CM, Vettore MV (2013). Gender differences in the association of perceived social support and social network with self-rated health status among older adults: a population-based study in Brazil. BMC Geriatr.

[17] Seedat S, Scott KM, Angermeyer MC (2009). Cross-national associations between gender and mental disorders in the World Health Organization World Mental Health Surveys. Arch Gen Psychiatry.

[18] KFF The independent source for health policy research, polling, and news Opioid Overdose Deaths by Race/Ethnicity 2021 Available at: https://www.kff.org/other/state-indicator/opioid-overdose-deaths-by-raceethnicity/?currentTimeframe=0&sortModel=%7B%22colId%22:%22Location%22,%22sort%22:%22asc%22%7D. Accessed January 1, 2024

[19] Burke MA, Sullivan R (2022). Can treatment with medications for opioid use disorder improve employment prospects?. Evidence from Rhode Island Medicaid enrollees.

[20] Tsemberis S, Gulcur L, Nakae M (2004). Housing First, consumer choice, and harm reduction for homeless individuals with a dual diagnosis. Am J Public Health.

[21] Watson DP, Shuman V, Kowalsky J (2017). Housing First and harm reduction: a rapid review and document analysis of the US and Canadian open-access literature. Harm Reduct J.

